# Medical Opinion and Movement

**Published:** 1906-07-21

**Authors:** 


### MEDICAL OPINION AND MOVEMENT.

AT a largely attended meeting, on July 11th, of the Governors of the City Orthopædic Hospital, it was resolved, by a large majority, that this hospital amalgamate with the National and Royal Orthopædic Hospitals on the terms suggested by King Edward's Hospital Fund for London.

A BILL has been introduced by the Home Secretary—Public Health (Regulations as to Food) Bill —for the purpose of entrusting the Local Government Board with power to make regulations for the prevention of danger arising to public health from the importation, storage, or distribution of articles of food.

THE importance of separating pure cases of coryza from those complicated by sore throat has been shown in statements recently advanced by Professor Prosser White and the experience of Drs. Arkwright and Allen. The former stated that "all who have worked at the bacteriology of a common cold have so invariably found the *bacillus septus* present at some stage of the illness." This view, it is contended, is not correct, for the collected experimental evidence available shows that this bacillus is only present where a sore throat complicates the coryza. We are clearly of opinion that it is of the first importance to keep pure cases of coryza isolated and apart from those which exhibit symptoms of sore throat.

THE holiday of the medical officer of health and the question of the payment of substitutes exercises an undue influence on some people's understanding. A man must have his holiday, and some large establishments insist on their employés taking it, because it affords an opportunity to ascertain how the work is really done and what is its quality. Our local authorities should arrange among themselves to have a staff of locums specially prepared for holiday duty. They might then be sometimes agreeably or otherwise surprised to hear the criticisms occasionally made by the more highly educated and younger men. Let every medical officer of health have his holiday each year, and let him be provided was a paid substitute during his absence.

MR. HALDANE, M.P., speaking on medical science to the students of the London Hospital, said in referring to the human body: "We knew that the parts of the organism were more like the citizens of a state than like the pieces of a machine. We now saw, for instance, that things which we took to be mere evils in themselves were really beneficent manifestations of the power of the living organism to throw off the poison which affected it. The new conception of medicine and surgery was to let nature alone as far as possible, and above all to assist in her work. Instead of being only one science, medicine was now one of many branches, and the problems to be solved were being worked at to-day in a fashion to which our forefathers were strangers. The surgeon, the physician, and the nurse required science to-day in a way in which they never required it before, and science had influenced and affected profoundly their whole teaching."

AN interesting point has been raised by Dr. T. Arthur Helme in advocating that some measures should be adopted in order to curtail the increasing expenditure of the British Medical Association. The Lancashire and Cheshire branch has issued a memorandum on the subject, which has been unanimously approved by the annual meeting of the South-Eastern Counties Division (Edinburgh branch). The memorandum referred to concerns the receipts and expenditure of the Association. It is stated that during 1905 the journal earned £23,500 by advertisements, sundry sales, etc., whilst the cost of production was £29,000, and the difference of £5,500 is treated as a loss. Of this expenditure £9,000 was for paper, £9,000 for printing, and £4,000 for postage and wrappers; the remaining £7,000 was expended in the preparation of literary matter and the collection of advertisements, including the cost of the staff. It further appears that the Association receives £25,000 a year from members' subscriptions. Dr. Radcliffe Crocker, the Treasurer of the Association, in reply to Dr. Helme's circular, points out that, as the organ of the Association, the journal expends at least £1,500 in printing matter for the information of members, which no paper privately owned would publish. The postage and special production of the colonial edition involve a serious loss, and further loss is experienced by the rejection of advertisements belonging to a large and highly remunerative class, many, if not all, of which would be published by a medical journal of the highest class run for profit only. Dr. Radcliffe Crocker admits it to be true that the journal during 1905 was not self-supporting from advertisements and sales to non-members to the extent of £5,500, but contends it is unjust to describe this sum as a loss, and to ignore the £25,000 received from members' subscriptions. If the advertisements excluded were admitted the so-called loss could easily be converted into a surplus, especially if analyses and notices of certain classes of products were inserted in the body of the journal, as is not unusual in similar publications. He further contends that each member gets the journal at a cost of less than 1½d. per week, post free, instead of paying double or quadruple this sum for an independent journal. These figures are instructive and interesting in many ways. Without being contentious, we may properly point out that the treasurer's communication gives force and point to Dr. T. Arthur Helme's endeavours to put the Association on a sound financial basis.

A FATAL case of plague is said to have occurred at Havre.

